# Vertical fluxes of nutrients enhanced by strong turbulence and phytoplankton bloom around the ocean ridge in the Luzon Strait

**DOI:** 10.1038/s41598-020-74938-5

**Published:** 2020-10-21

**Authors:** Eisuke Tsutsumi, Takeshi Matsuno, Sachihiko Itoh, Jing Zhang, Tomoharu Senjyu, Akie Sakai, Keunjong Lee, Daigo Yanagimoto, Ichiro Yasuda, Hiroshi Ogawa, Cesar Villanoy

**Affiliations:** 1 Atmosphere and Ocean Research Institute, University of Tokyo, Kashiwa, 277-8564 Japan; 2 Research Institute for Applied Mechanics, Kyushu University, Kasuga, 816-8580 Japan; 3 Graduate School of Science and Engineering, University of Toyama, Toyama, 930-8555 Japan; 4 Interdiciplinary Graduate School of Engineering Science, Kyushu University, Kasuga, 816-8580 Japan; 5 Marine Science Institute, University of the Philippines, 1101 Metro Manila, Philippines

**Keywords:** Ocean sciences, Marine biology, Physical oceanography

## Abstract

Steep oceanic ridges and tidal currents in the Luzon Strait generate some of the world’s strongest turbulent mixing. To evaluate the impacts of the turbulence intensity on the marine ecosystem, we carried out measurements of microstructure turbulence and biogeochemical hydrography along 21°N in the Luzon Strait during the R/V *Hakuho Maru* cruise, KH-17-5-2, in November 2017. We found a turbulent kinetic energy dissipation rate exceeding *O*(10^−7^) W kg^−1^ and vertical eddy diffusivity exceeding *O*(10^−3^) m^2^ s^−1^, two orders of magnitude larger than those in the open ocean, above a shallow sub-ridge on the eastern ridge of the Luzon Strait. In addition, a clear chlorophyll *a* bloom was identified in the surface layer above the sub-ridge from in situ measurements and satellite observations. High values of nitrate (4.7 mmol N m^−2^ d^−1^) and phosphate (0.33 mmol P m^−2^ d^−1^) fluxes estimated near the base of the surface chlorophyll *a* bloom strongly suggest that enhanced turbulent mixing promotes nutrient supply to the euphotic zone and generates new production within the surface layer, contributing to the formation of a quasi-permanent local chlorophyll *a* bloom north of Itbayat Island on the eastern ridge.

## Introduction

The Kuroshio is the western boundary current (WBC) forming part of the North Pacific sub-tropical gyre, transporting heat and material from low to high latitudes. Recent studies have revealed that strong vertical mixing is generated in certain areas of the WBC region. For example, within the Kuroshio, elevated turbulent kinetic energy (TKE) dissipation rate *ε* and vertical eddy diffusivity *K*_*ρ*_ have been identified in the Tokara Strait where the Kuroshio enters the northwestern Pacific^1,2^, in the Shikoku Basin south of Japan^3^ and in the Green Island area east of Taiwan^4^. These studies suggested that the strong vertical mixing observed was caused by interactions between the strong Kuroshio current and the abrupt topography, such as shallow seamounts, ocean ridges, and shelf breaks. The topographic depths are 100–200 m deep, comparable to the typical depth of the euphotic zone in the Kuroshio. Therefore, it can be expected to affect biological productivity in the Kuroshio through the vertical transport of nutrients to the sunlit layer^3^. Kobari et al.^5^ recently suggested that phytoplankton productivity may be stimulated by turbulent nitrate flux amplified within the Kuroshio in the Tokara Strait and rapidly transferred to microzooplankton through grazing.

The Luzon Strait is an area where strong vertical mixing occurs in the Kuroshio path. The two dominant ocean ridges in the Luzon Strait, the eastern and western ridges, generate strong internal waves and turbulent mixing associated with tidal motion, topographic resonance, and the Kuroshio^6–11^. Above the western ridge, Alford et al.^12^ estimated *ε* and *K*_*ρ*_ from a vertical scale of density overturn, the Thorpe scale^13,14^, and found that they reached extremely large values at depths of 500–1000 m of *O*(10^−6^) W kg^−1^ and *O*(10^−1^) m^2^ s^−1^, respectively. A large eddy simulation by Jalali and Sarkar^15^ confirmed that generation of the strong turbulence is associated with lee waves as well as downslope jets, critical slope bottom boundary layers, and high mode internal wave beams, all of which are induced by tide–topography interactions. At the eastern ridge, observations and numerical simulations indicate that the breaking of tidally generated lee waves leads to density overturns of ~ 200-m height in the bottom 500–1000 m depth^6,16^. The associated overturn-based vertical eddy diffusivity reaches *O*(10^−1^) m^2^ s^−1^, similar values to those observed at the western ridge^12^. Mensah et al.^17^ applied a bulk heat balance to estimate mean vertical diffusivities of *O*(10^−4^) m^2^ s^−1^ in the relatively shallow layers (approximately 70–250 m depth) over the Kuroshio path in the Luzon Strait using historical hydrographic observations spanning three decades.

While many investigators have identified strong turbulence in the Luzon Strait, few studies have investigated turbulent mixing with direct measurements of turbulence microstructure^18^, as mentioned by Sun and Wang^19^. This approach gives a more accurate estimate of turbulence intensities than the overturn-based method, especially in the upper ocean where stratification is strong and therefore vertical scales of density overturn tend to become small. Microstructure measurements have been carried out in the South China Sea (SCS) where internal tides and/or waves generated in the Luzon Strait propagate and break^19–24^. Yang et al.^22,23^ showed elevated vertical eddy diffusivity of *O*(10^−4^–10^−3^) m^2^ s^−1^ in the pycnocline and its relation to the intruded Kuroshio or mesoscale eddies. St. Laurent^20^ presented intensified TKE dissipation associated with nonlinear internal waves around the shelf break in the northern SCS. Sun et al.^24^ found high vertical eddy diffusivity of *O*(10^−4^) m^2^ s^−1^ in the pycnocline near the Luzon Strait and the Dongsha Plateau, which is likely induced by internal waves.

In addition to the above studies, research has highlighted the effects of the internal-wave-induced mixing on marine biological productivity in the Luzon Strait/SCS region. Jan et al.^25^ suggested the potential effects of internal tides generated in the Luzon Strait on biogeochemical environments on the southern coast of Taiwan, focusing on vertical advection of nutrients and vertical mixing induced by shoaling internal tides. Wang et al.^26^ suggested that internal waves could affect phytoplankton blooms at the Dongsha Atoll through upwelling of organic matters and subsequent microbial regeneration. Du et al.^27^ identified vertical turbulent nutrient fluxes in the summertime SCS based on microstructure measurements, showing their dominant role in supporting primary production within the subsurface nutrient replete layer. Many studies have revealed high biological productivity from the western Luzon Strait through the SCS despite its traditional classification as an oligotrophic area (e.g. Ref.^28^). Phytoplankton blooms are typically observed during wintertime, called Luzon winter blooms. Such blooms are characterized by an inverted-v shape formed along fronts between the Kuroshio and SCS water or the Luzon coastal current^29,30^. The winter bloom is thought to be caused by nutrient supply associated with processes including frontal upwelling, eddy-induced upwelling, monsoon-induced upwelling, and freshwater from rivers such as the Cagayan River on the northern coast of Luzon Island^29–33^. Shang et al.^32^ suggested that the vertical mixing associated with the upwelling plays a role in sustaining the phytoplankton blooms.

**Figure 1:**
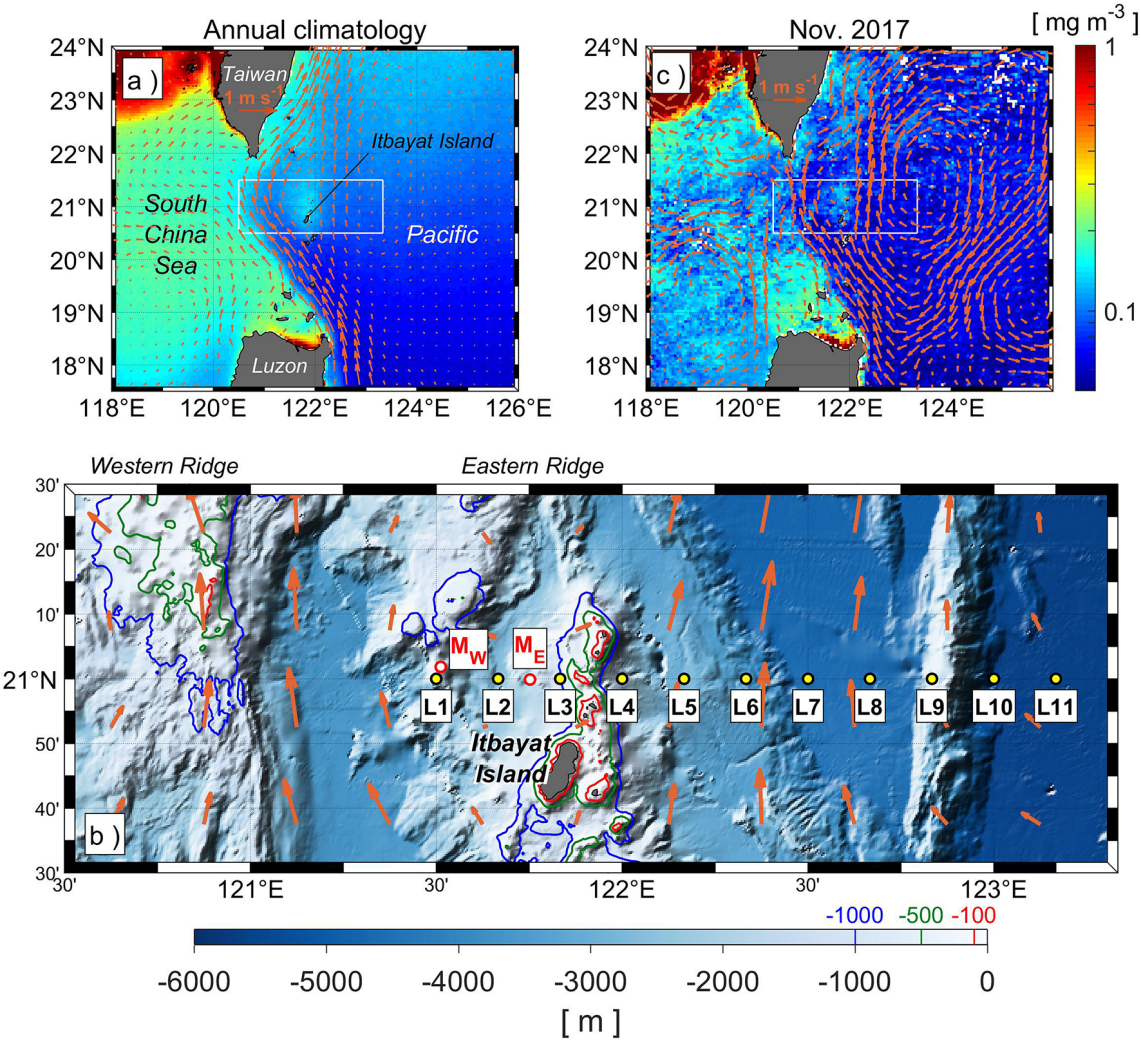
Map of surface chlorophyll *a* concentration, geostrophic currents, and bathymetry around the Luzon Strait. (**a**) Annual climatology of MODIS-Aqua chlorophyll *a* (color shading) and CMEMS absolute geostrophic velocity (arrows) during 2004–2018. The location of the experiment site is indicated by the white rectangle. (**b**) Bathymetric map with microstructure stations L1–L11. Red circles represent mooring stations; M_W_ and M_E_ are where upward-looking 75-kHz Long Ranger ADCPs were moored. Red, green, and blue lines in the bathymetric map show the 100, 500, and 1000-m isobaths, respectively. Arrows represent absolute geostrophic velocity during the experiment period. (**c**) Weekly averaged surface chlorophyll *a* and absolute geostrophic velocity, as in (**a**) but for November 2017. Color scale for chlorophyll *a* is the same in (**a**) and (**c**).

Compared to the progress in the physical and biogeochemical oceanography of the region from the western Luzon Strait to the SCS, knowledge about upper-ocean mixing and its effects on biogeochemical fields is limited in the eastern Luzon Strait. Chen et al.^34^ found moderate but sustained phytoplankton blooms in the surface Kuroshio water near the Luzon Strait associated with new production from nitrate uptake in winter and from nitrogen fixation in summer. In fact, such annual blooms can be detected within the Kuroshio in the climatology of satellite-derived chlorophyll *a* (MODIS-Aqua), especially around the small islands north of Itbayat Island on the eastern ridge (Fig. 1a,b). The blooms are clearly separated from relatively high chlorophyll *a* concentration in the SCS by the Kuroshio and may have a role in biological productivity in the oligotrophic Kuroshio waters (Fig. 1a), although few studies have investigated this issue in detail.

Here, we present surveys of turbulence microstructure and biogeochemical hydrography conducted in the Luzon Strait in late-autumn/early-winter of 2017 and reveal the detailed structure of upper-ocean turbulence intensities and chlorophyll *a* concentration around the eastern ridge of the Luzon Strait. Weekly averaged MODIS Aqua chlorophyll *a* data suggest the occurrence of a local chlorophyll *a* bloom north of Itbayat Island, where we performed observations along a zonal section and set moorings during a field experiment (Fig. 1c). Because primary production is likely attributed to new production by nutrient uptake in our experimental area/period^34^, this study investigates a possible linkage between topographically enhanced turbulent mixing and the chlorophyll *a* bloom north of Itbayat Island through nutrient supply from depth. The elevated nutrient fluxes occurred within or on the Pacific side of the Kuroshio, suggesting that these fluxes possibly fertilize the Kuroshio and/or North Pacific tropical waters.

## Results

### Observations

We carried out measurements of turbulence intensity at 11 stations (L1–L11) along a zonal section between 121°30′E and 123°10′E, 21°N in the Luzon Strait (Fig. 1b). The eastern ridge of the strait is in the western part of the section and there is a shallow ocean sub-ridge between L3 and L4. The survey was conducted during the R/V *Hakuho Maru* cruise KH-17-5-2 in November 2017. On 22–23 November, turbulence measurements with the TurboMAP-L (hereinafter, TM) profiler were performed at three stations (L3, L4, and L5). After a brief hiatus due to rough weather conditions, measurements were made at ten stations (L1–L10) on 25–26 November. Turbulence measurements with the VMP-2000 (VMP) profiler were performed at three stations (L1, L2, and L11) on 22 and 25 November. We use the data obtained on 25–26 November with the microstructure profilers in the analysis below. From 22 to 28 November, we measured temporal variations in current fields with moored acoustic Doppler current profilers (Workhorse Long Ranger ADCP, Teledyne Marine, USA) at M_W_ (close to L1) and M_E_ (between L2 and L3) (Fig. 1b). The vertical bin size is 8 m and the ensemble interval is 1 min. Vertical profiles over depths of 66–602 m and 56–504 m were obtained at stations M_W_ and M_E_, respectively. Vertical profiles of temperature and salinity were also obtained with shipboard CTD (SBE-9plus, SeaBird Scientific Co.) at stations L1, L2, L4, L8, and L11, and with expendable CTD (XCTD, Tsurumi-Seiki Co.) at stations. L1, L5, L6, L7, L9, and L10. At the shipboard CTD stations, we took water samples for nutrient (nitrate, nitrite and phosphate) and chlorophyll *a* analysis.

### Water-mass structure

**Figure 2:**
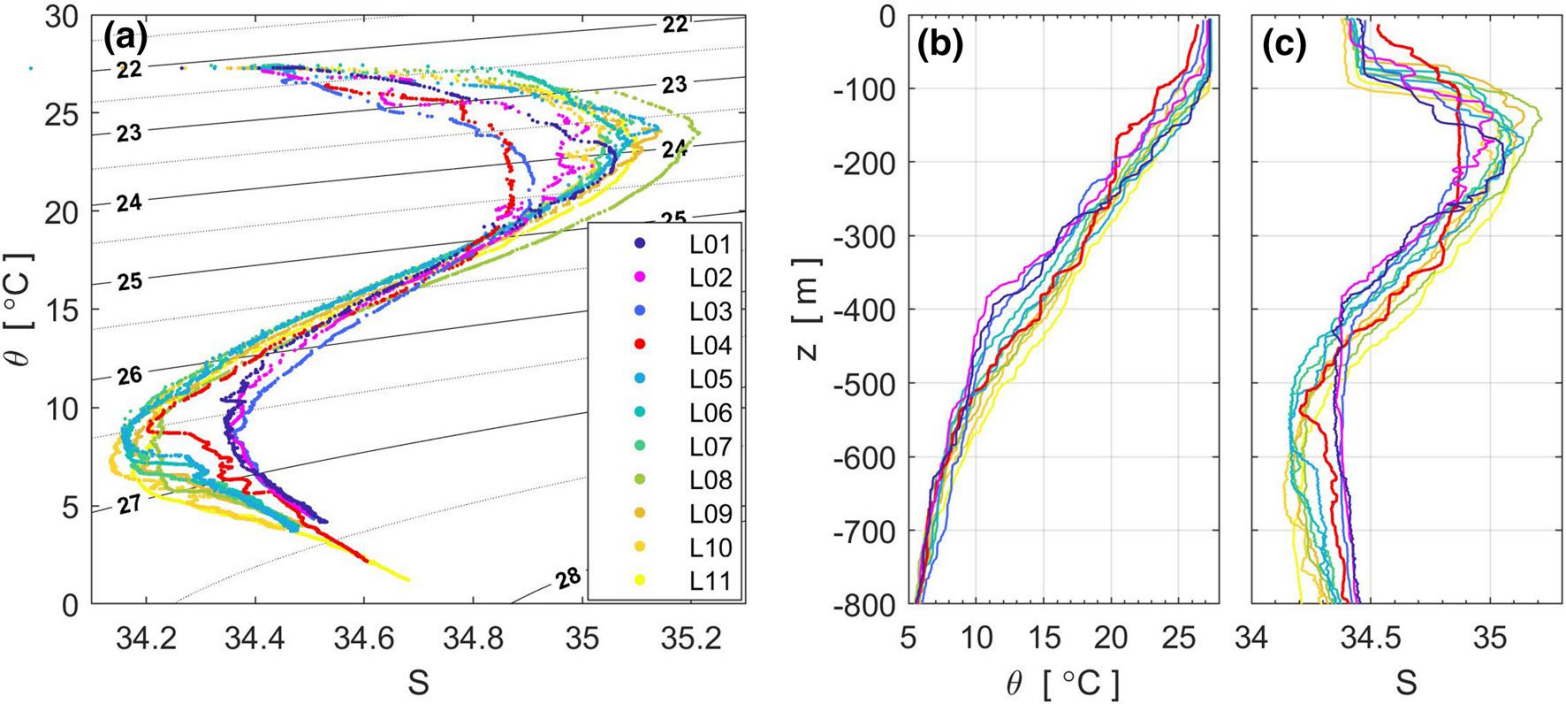
Potential temperature–Salinity (*θ*–S) structure from shipboard CTD and XCTD along the survey line. (**a**) *θ*–S diagram, (**b**) potential temperature profiles, and (**c**) salinity profiles. Contour lines in panel (**a**) denote potential density at 0.5 kg m^−3^ intervals.

Around the Luzon Strait, the vertical structure of potential temperature *θ* and salinity shows a clear salinity maximum (minimum) in the subsurface (deeper) layers (Fig. 2a), a common structure in the northwestern Pacific (e.g., Mensah et al.^17^). Salinity maxima seen around potential density (σ_θ_) ~ 24.0 were 34.8–34.9 at L3 and L4, 0.2–0.3 lower than those of the Kuroshio tropical water found at the other stations (L1, L2, L5–L10). Temperature and salinity are relatively homogeneous within 170–230 m layer at L4 (Fig. 2b,c) implying that the less saline water is likely caused by the strong vertical mixing noted in the following section. Mensah et al.^17^ suggested that strong mixing in the Luzon Strait can reduce in the subsurface salinity maximum of the Kuroshio tropical water. However, salinity minima around σ_θ_ ~ 26.5, which is roughly equivalent to 500–600 m depth, are ~ 0.2 more saline at the western three stations L1–L3 than at the other stations. The saline salinity minima likely originate from the South China Sea Intermediate Water, which is characterized by salinity minima ~ 34.41 at 500–600-m depth^35^, rather than being locally generated by vertical mixing. Since this study focuses on vertical mixing in the upper ocean in the Luzon Strait, we mainly consider the mixing around the salinity maximum between 100 and 250 m.

### Turbulent mixing

**Figure 3:**
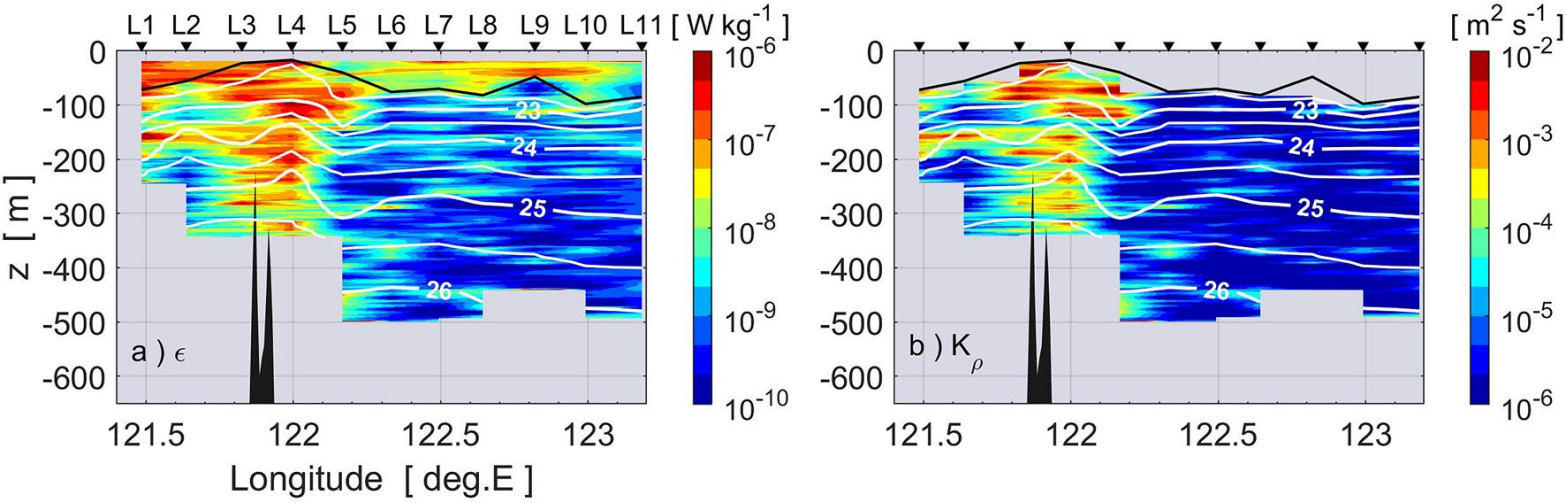
Vertical section of (**a**) TKE dissipation rate *ε* and (**b**) vertical eddy diffusivity *K*_*ρ*_ estimated by TurboMAP-L and VMP-2000 profilers from L1 to L11 on 25–26 November, 2017. Contour lines show isopycnals of potential density (σ_θ_). The thick solid line indicates the base of the surface mixed layer, where potential density deviates by 0.03 kg m^−3^ from the uppermost density observations. Dark shading represents the bottom topography along the ship track from a shipboard sounder.

**Figure 4:**
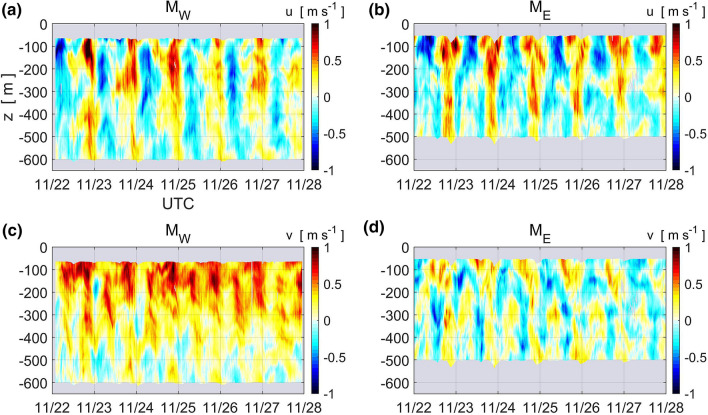
Temporal variations in the vertical structure of current velocity obtained by moored ADCP at M_W_ (**a**, **c**) and M_E_ (**b**, **d**). Zonal and meridional velocities are shown in the upper (**a**, **b**) and lower (**c**, **d**) panels, respectively.

The vertical section of the TKE dissipation rate *ε* along 21°N identified by microstructure profilers is shown in Fig. 3a. Below the surface mixed layer, *ε* was elevated to ≥ 10^–7^ W kg^−1^ around the shallow sub-ridge, particularly at L3 and L4, compared to the open ocean value of 10^–8^ W kg^−1^ east of L6. As seen in the temporal variations in current velocity obtained at the mooring sites west of the sub-ridge, diurnal tidal currents were predominant in the eastward component at both mooring sites (Fig. 4a,b), while a continuous northward current was observed at station M_w_ (Fig. 4c), which is likely a branch of the Kuroshio (Fig. 1c). This suggests that the enhanced turbulence may be caused by interactions between the tidal motion and the steep bottom topography. In fact, shipboard expendable bathythermograph (XBT) and shipboard ADCP observations indicate active internal waves around the sub-ridge during the cruise (Sakai et al., manuscript submitted to *Journal of Oceanography*). The large value of *ε* as well as the above-mentioned decreased subsurface salinity maxima suggest strong vertical mixing at the sub-ridge. This is evident in the vertical eddy diffusivity *K*_*ρ,*_which is also elevated above the ridge to ≥ 10^−3^ m^2^ s^−1^, while it is smaller than ≤ 10^−5^ m^2^ s^−1^ in the eastern part of the section (Fig. 3b).

### Vertical turbulent nutrient fluxes

**Figure 5:**
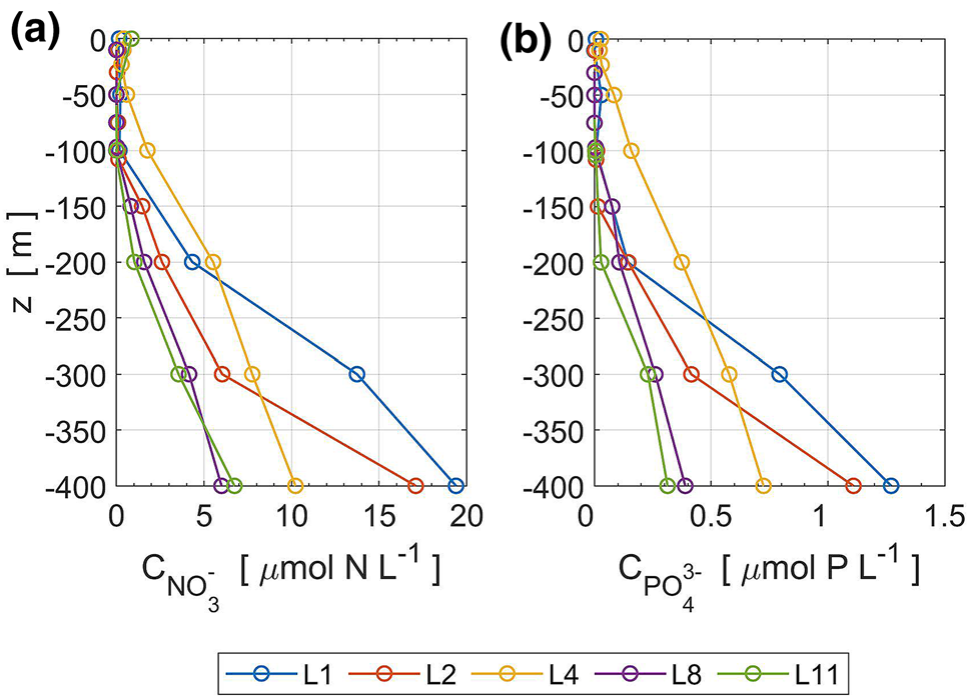
Vertical profiles of (**a**) nitrate and (**b**) phosphate obtained from water sampling at stations L1, L2, L4, L8, and L11 on 25–26 November.

The intensified vertical mixing found close to the sub-ridge enhances vertical transport of material there. Vertical profiles of the nutrients (Fig. 5) show that nitrate and phosphate are largely depleted in the surface mixed layer. Nitrate and phosphate concentrations are relatively greater in the surface 200-m layer at station L4 than at other stations, implying shoaling of the nutricline. The *θ*–S curve at L4 implies vertical mixing at depths of 100–200 m (Fig. 2) and enhanced vertical mixing was detected at L4; this suggests that shoaling of the nutricline occurred at station L4 likely due to strong vertical mixing.

**Figure 6:**
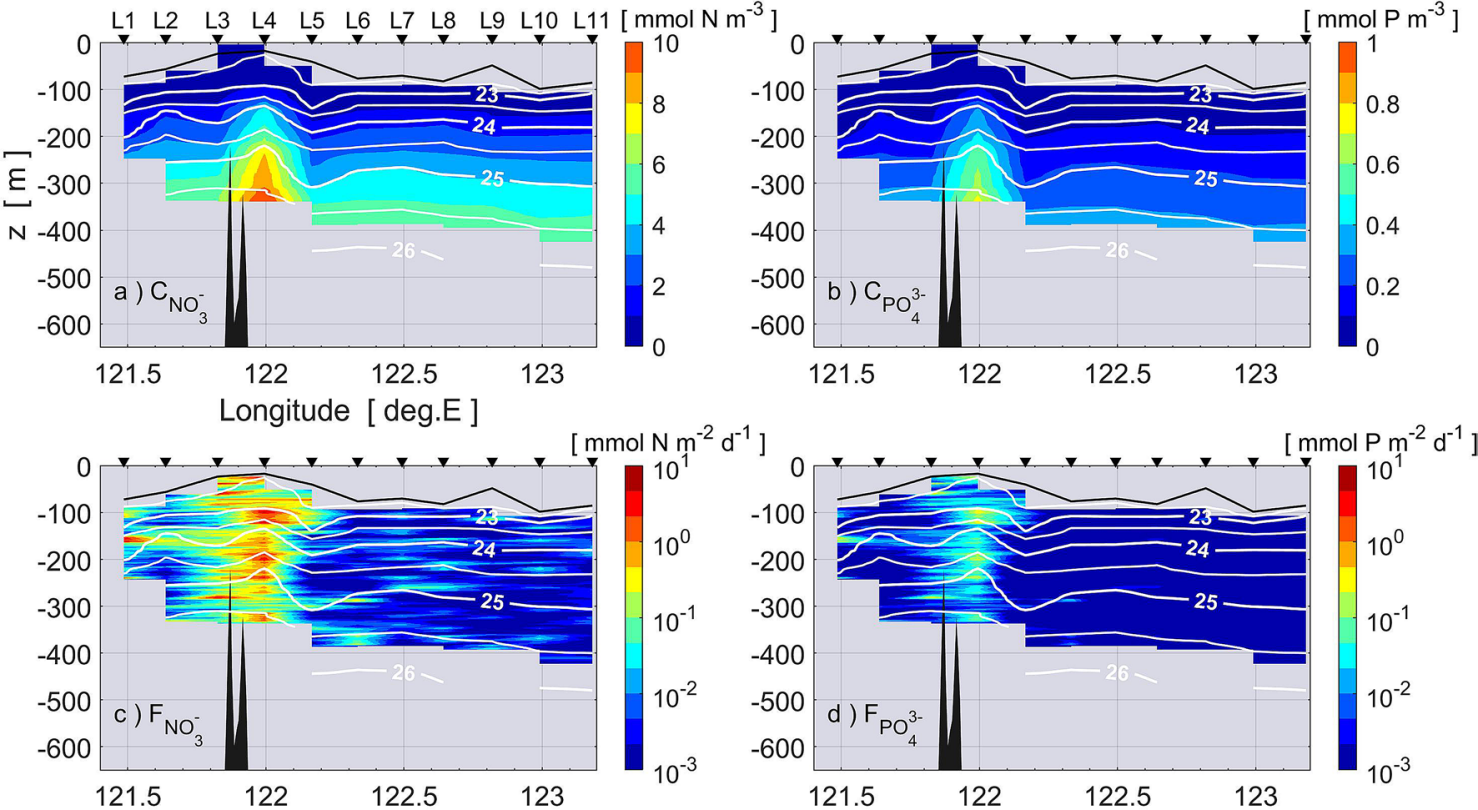
Vertical section of nutrients and vertical turbulent nutrient fluxes: (**a**) nitrate concentration, (**b**) phosphate concentration, (**c**) vertical turbulent nitrate flux, (**d**) vertical turbulent phosphate flux. Nutrient concentrations were estimated from potential temperature within a range of 15–27 °C. Contour lines show isopycnals and the thick solid line represents the depth of the surface mixed layer.

**Figure 7:**
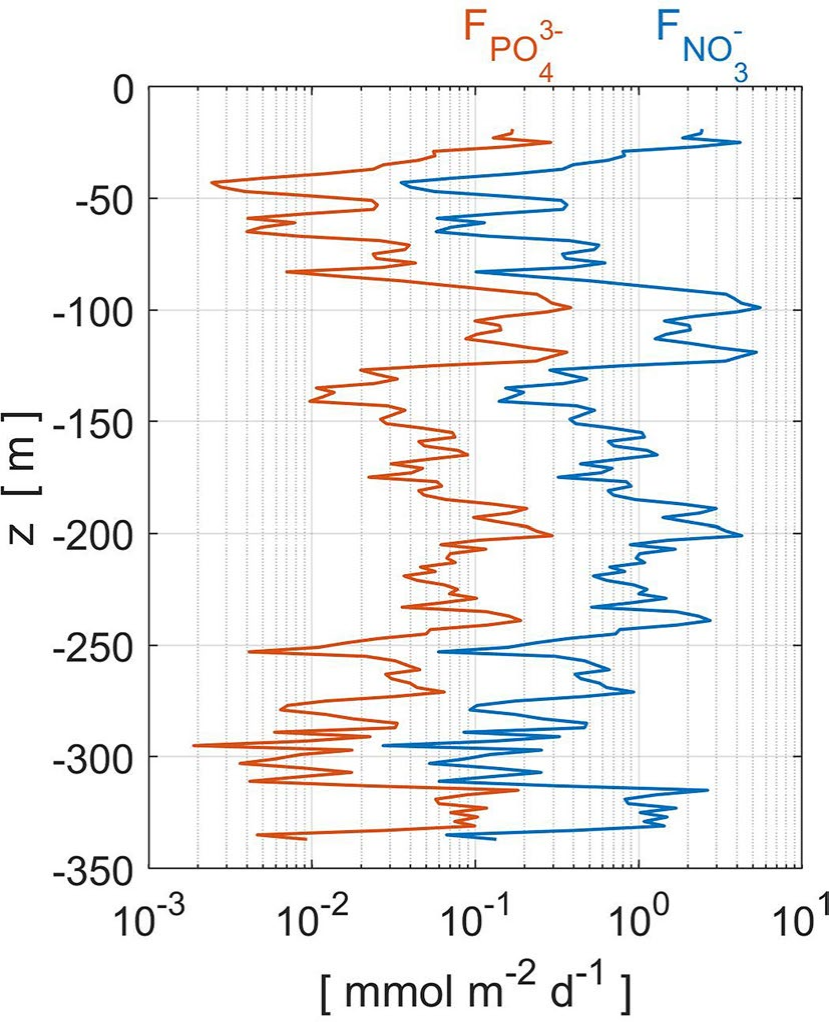
Vertical profiles of vertical turbulent flux of nitrate (blue) and phosphate (red) estimated at station L4.

Figure 6a and b show vertical sections of nitrate and phosphate, respectively, estimated using potential temperature from the microstructure profiler by linear regression (see Methods). Vertical turbulent fluxes of nitrate and phosphate are estimated using the vertical gradient of nitrate and phosphate along with the vertical eddy diffusivity [see Eq. (1) in “Methods” section], which are shown in Fig. 6c and d, respectively. As expected, a much larger vertical flux of nitrate is seen around the sub-ridge than on the open ocean side. The maximum value of the nitrate (phosphate) flux was found at L4, reaching 4.7 mmol N m^−2^ d^−1^ (0.33 mmol P m^−2^ d^−1^) at around 100-m depth (Fig. 7). Because the typical thickness of the euphotic zone is 100 m in this region^34^, we hypothesize that the enhanced vertical fluxes of the nitrate and phosphate may contribute to the primary production in the upper layer.

### Surface chlorophyll *a* bloom

**Figure 8:**
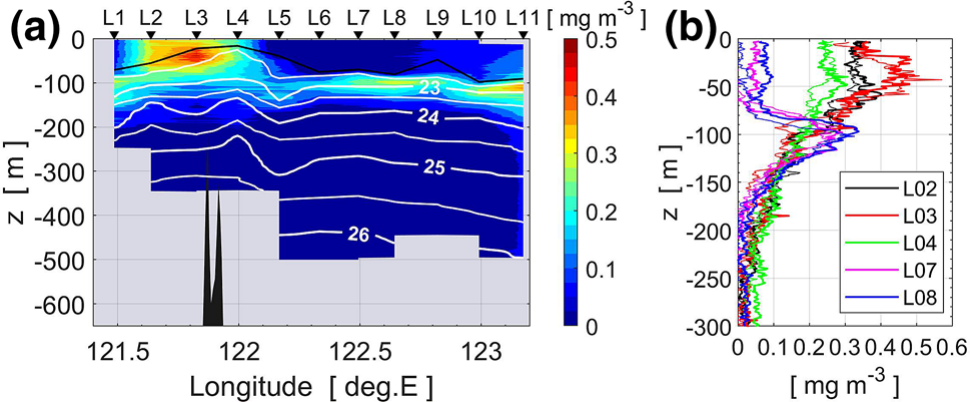
(**a**) Vertical section of chlorophyll *a* obtained by the TurboMAP profiler from L1 to L10 and at L11 by SBE-9plus on 25–26 November. Contour lines show isopycnals and the thick solid line represents the surface mixed layer depth. (**b**) Vertical profiles of chlorophyll at L2, L3, L4, L7, and L8, where thick and thin lines show profiles obtained with the two casts at each station.

The vertical section of chlorophyll *a* concentration (Fig. 8a) shows that in the eastern part of the section (L5–L11) there is a clear subsurface chlorophyll *a* maximum around 100-m depth. On the other hand, a strong chlorophyll *a* bloom was found over waters shallower than 100 m above the sub-ridge (stations L2, L3, and L4), with a concentration of 0.2–0.5 mg m^−3^ and a zonal scale of roughly 50 km. The size and position of the surface bloom are nearly consistent with those of the local surface bloom north of Itbayat Island detected in the MOIDS-Aqua chlorophyll *a* during the experimental period (Fig. 1c). Therefore, the surface chlorophyll *a* bloom observed by the in situ measurement is likely the same as the local chlorophyll *a* bloom north of Itbayat Island.

## Discussion

From surveys of turbulence microstructure and biogeochemical hydrography, we found elevated vertical eddy diffusivity of *O*(10^−3^) m^−2^ s^−1^ and large vertical nitrate flux of *O*(1) mmol N m^−2^ d^−1^ at around 100-m depth above shallow topography on the eastern ridge of the Luzon Strait in late-autumn/early-winter (Fig. 3b–d). We also observed a surface chlorophyll *a* bloom above the strong turbulent nutrient flux (Fig. 8). According to Chen et al.^34^, in the eastern Luzon Strait the ratio of nitrate-uptake-based new production to primary production is approximately 0.5 in winter and 0.3 in summer within the euphotic zone of the Kuroshio water, indicating that a significant part of the primary production is supported by the nitrate-uptake-based new production throughout the year. Because strong turbulent mixing is highly likely driven by tides in the Luzon Strait, the enhancement of the turbulent nitrate flux would be regulated above the shallow sub-ridge. Therefore, we conclude that enhanced turbulent mixing above the shallow sub-ridge likely sustains a significant part of the surface chlorophyll *a* bloom north of Itbayat Island.

The vertical extent of the layer of strong turbulence was limited at station L1 compared to that at L4, resulting in the formation of a chlorophyll *a* maximum in the subsurface at L1 around 100–120 m depth (Fig. 8a). The lower chlorophyll *a* concentration in the upper layer at station L1, despite the relatively large mixing, could be caused by the influence of the Kuroshio, which passes through the Luzon Strait (Fig. 1b,c). Station L1 was located around the southern flank of another sub-ridge on the eastern ridge where there was high turbulence intensity at the mid-depths (Fig. 3), and consequently there were large nutrient fluxes (Fig. 6). However, the path of the Kuroshio in the Luzon Strait varies considerably, and during our observation the Kuroshio appeared to flow around the western ridge and far east of the eastern ridge (Figs. 1c and 4). The Kuroshio flowing northward around L1 might sweep the nutrients downstream, so that nutrients were not able to accumulate and enable growth of phytoplankton.

There is a slight discrepancy in the location of elevated nutrient flux and chlorophyll *a* (Fig. 6c,d and 8a). This discrepancy can be explained as follows. Nutrient fluxes estimated from the intensity of vertical mixing and nutrient gradient are primary driven by simultaneous current velocity shear, mainly caused by internal waves associated with tidal motion. On the other hand, chlorophyll *a* concentration has a couple day delay in reaching its maximum as the phytoplankton grow. The largest vertical mixing may occur around L4 or around L3 depending on the tidal phase. However, averaged over a sub-tidal or longer timescale, large vertical transport of nutrients can be expected around the sub-ridge. The primary production above the sub-ridge in some sense represents an integration of the vertical fluxes of nutrients. Once the high chlorophyll *a* region is formed above the sub-ridge, it may be advected laterally by tidal currents. Measurements at stations L4 and L3 were carried out in the early afternoon on 25 November, just before westward tidal current changed from westward to eastward. This is consistent with the fact that the core of the chlorophyll *a* patch was found on the western edge of the sub-ridge, at station L3.

Here, we discuss the possible relationship between the turbulent nutrient transport to the surface layer and the surface chlorophyll *a* bloom. If we assume that nitrogen supplied by turbulent flux comes from nitrate, and all of it is used for new production in the surface layer, it is possible to estimate the amount of organic carbon to be generated by the new production. Assuming that the euphotic zone occupies the surface 100 m at our experimental region^34^, we calculate the mean nitrate-uptake-based new production within the surface 100-m using the Redfield ratio, *r*_C-N_ = 106/16 and the atomic weight of carbon, M_c_ = 12. Thus, the observed vertical nitrate flux at 100-m depth, 4.7 ± 1.9 mmol N m^−2^ d^−1^, gives an estimate of the new production integrated over the surface 100-m layer, $M_{c} \,r_{C - N} F_{{NO_{3}^{ - } }} =$ 0.37 ± 0.15 g C m^−2^ d^−1^, where the error estimate considers the uncertainty in the regression between nitrate concentration and water temperature (see Methods). This estimate is based on the premise that the limiting factor for new production is nitrogen. Similarly, we consider the phosphorus-limited condition with maximum vertical phosphate flux at station L4, 0.33 ± 0.16 mmol P m^−2^ d^−1^, and the Redfield ratio for phosphorus, *r*_C-P_ = 106/1, leading to an estimate of phosphate-based new production, 0.42 ± 0.20 g C m^−2^ d^−1^, slightly higher than the estimate based on nitrogen. Since the observed ratio of nitrogen to phosphorus was smaller than the Redfield ratio (16), nitrogen appears to be the limiting factor. The indirect estimates of the nitrogen-based integrated new production within the euphotic zone are reasonably consistent with an estimate from in situ measurements around the wintertime Luzon Strait by Chen et al.^34^, 0.27 g C m^−2^ d^−1^, supporting our conclusion.

Furthermore, we try to estimate the depletion rate of chlorophyll *a* in the steady-state, *γ*, by equating the nitrogen-based chlorophyll *a* production rate to the depletion,

$$ M_{c} \,r_{C - N} r_{C - Chl}^{ - 1} F_{{NO_{3}^{ - } }} = \gamma h_{e} \overline{C} , $$

where *r*_C-Chl_ is the carbon to chlorophyll *a* ratio and $\overline{C}$ is the depth-averaged concentration of chlorophyll *a* in the euphotic layer with a thickness *h*_*e*_ = 100 m. The carbon to chlorophyll *a* ratio varies considerably^36,37^, so we used a range of values, *r*_C-Chl_ = 25–52, based on in situ measurements for pico-phytoplankton, which dominate chlorophyll *a* concentration in the wintertime Luzon and SCS^38^. Using the observed value above the sub-ridge, $\overline{C}$ = 0.4 mg m^−3^, we obtain *γ* = (0.16 ± 0.06)–(0.33 ± 0.13) d^−1^. This indicates that 16–33% of chlorophyll *a* is lost in one day. It is noted that because we assume an equilibrium state between phytoplankton production (or, growth) rate and its mortality, the estimated *γ* equivalently means a phytoplankton growth rate of 0.16–0.33 d^−1^. This estimate does not account for the effect of regenerated production. Although our experiment cannot provide any estimate of regenerated production, a ratio of ~ 0.5 of nitrate-uptake-based new production to primary production in the wintertime Kuroshio surface water near the Luzon Strait from Chen et al.^34^ gives a modified estimate, *γ* = (0.32 ± 0.06)–(0.66 ± 0.26) d^−1^.

These discussions are based on the assumptions of a steady-state balance between vertical nutrient transport and its consumption by phytoplankton and an equilibrium state between phytoplankton growth and losses over the sub-ridge. However, if horizontal advection has a significant impact on the nitrate concentration over the sub-ridge, the former assumption would fail and dynamic interplay between phytoplankton growth and losses would have roles on the formation of the surface chlorophyll bloom. Since mean currents such as the Kuroshio was very weak around the sub-ridge, the effect of the horizontal advection may be evaluated with a ratio of tidal excursion length, *L*_*e*_ to horizontal topographic length scale (or half width of the strong turbulence area), *W*: if *L*_*e*_ is much larger (smaller) than *W*, then horizontal advection would have a major (minor) impact on the surface bloom formation. Based on the current observation at Stn.M_E_, current in the surface 200-m layer is dominated in the zonal direction and by diurnal tide (frequency: *ω*_K1_ = 7.3 × 10^−5^ rad s^−1^) with the amplitude *U*_K1_ = 0.5 m s^−1^ (Fig. 4b), which leads to *L*_*e*_ = *U*_K1_*ω*_K1_^−1^ =  ~ 7 km. This gives *L*_*e*_/*W* = 0.4–0.7 with *W* = 10–20 km, suggesting that horizontal advection may have a certain but not major role in the formation of the surface chlorophyll bloom.

Kobari et al.^5^ estimated high mortality rates of total phytoplankton due to grazing by zooplankton of 0.38–1.05 d^−1^ in the surface Kuroshio water from dilution experiments in the late-autumn Tokara Strait. The high mortality rates indicate rapid trophic transfer to zooplankton and suggest important roles of the topographically enhanced turbulent nutrient fluxes in the high biological productivity in the apparently oligotrophic Kuroshio^5^. They also estimated phytoplankton net growth rates of ≤ 0.55 d^−1^. In the Tokara Strait, turbulent mixing and turbulent nutrient flux are enhanced in the surface/subsurface Kuroshio water due to flow–topography interaction, similar our observations in the Luzon Strait. Although our estimate of the chlorophyll *a* depletion rate includes many assumptions, its similarity to the estimate by Kobari et al.^5^ may suggest a common role of the strong turbulent mixing in the Kuroshio water on higher trophic level productivity. However, similar but relatively small estimates of phytoplankton growth rate in this study (0.16–0.33 d^−1^) implies that factors not considered here, such as the horizontal advection process, may necessary to be considered. Because our observation is too sparse to resolve the advection effects and associated dynamic phytoplankton growth and loss interplay, further experiments are required for their detailed evaluation.

## Methods

### Microstructure data processing

Microstructure current velocity shear was measured using loosely tethered, free-falling profilers, TurboMAP-L (hereinafter, TM, manufactured by JFE Advantech, Japan) and VMP-2000 (VMP, manufactured by Rockland Scientific, Canada). Each profiler is equipped with two shear probes and sample velocity shear at 512 Hz. The TM profiler descends freely at about 0.6 m s^–1^ nominally down to 500-m depth, although at some stations it stopped at 250–350 m depth due to a limitation of the cable length and strong tension on the cable caused by strong shear current and/or fast ship drift under strong wind conditions. The VMP profiler makes measurements down to 1000-m depth. The TM profiler was cast twice and the VMP profiler once at each station. To estimate turbulent kinetic energy dissipation rate, $\varepsilon = 7.5\nu \overline{{\left( {{{\partial u^{\prime}} \mathord{\left/ {\vphantom {{\partial u^{\prime}} {\partial z}}} \right. \kern-\nulldelimiterspace} {\partial z}}} \right)^{2} }}$ (unit: W kg^–1^) where *ν* is the molecular dynamic viscosity of seawater*,* the variance of the microstructure shear was calculated by integrating the wavenumber spectrum with visual checking of spectral consistency between the semi-empirical Nasmyth spectrum and observed spectrum^39^. Vertical profiles of fluorescence and turbidity were also obtained by a fluorometer/optical backscatter sensor on the TM profiler. Since the two profiles obtained from the two casts at each station were roughly consistent, we used their average for analyses. Vertical profiles of vertical eddy diffusivity coefficient for density, *K*_*ρ*_ were calculated by the formula proposed by Osborn^40^, $K_{\rho } = \Gamma \varepsilon N^{ - 2}$ (unit: m^2^ s^–1^), with the assumption of constant mixing efficiency Γ = 0.2. Here, *N* is the Brunt–Väisälä frequency which is calculated from the resorted potential density *ρ*_*s*_ via $N^{2} = - \left( {{g \mathord{\left/ {\vphantom {g \rho }} \right. \kern-\nulldelimiterspace} \rho }_{0} } \right)\left( {{{\partial \rho_{s} } \mathord{\left/ {\vphantom {{\partial \rho_{s} } {\partial z}}} \right. \kern-\nulldelimiterspace} {\partial z}}} \right)$ where *g* is the gravitational acceleration and *ρ*_0_ is a reference density (1027.0 kg m^–3^). Note that *K*_*ρ*_ is not calculated within the surface mixed layer where Osborn’s hypothesis^40^ would fail.

### Estimation of vertical turbulent nutrient fluxes

The vertical turbulent flux of nutrients may be quantified as

1 $$ F = - K\frac{\partial C}{{\partial z}}\quad \left( {{\text{unit: mmol m}}^{ - 3} {\mkern 1mu} {\text{d}}^{ - 1} } \right), $$

where *C* is nutrient concentration and *K* is the corresponding vertical eddy diffusivity coefficient. In this study, we assume *K* = *K*_*ρ*_. Nutrient concentration data from bottle samples were sparse in both vertical and horizontal directions, so it would be difficult to make a reliable nutrient map along the vertical section with the nutrient data from bottle sampling. In this study, we estimate the vertical profile of nutrient concentrations, nitrate (NO_3_^–^) and phosphate (PO_4_^3–^), using their linear relationship with water temperature. We observed a positive linear correlation between nutrients and potential temperature, particularly in the upper layer where temperature is > 15 °C (Supplementary information, Fig. S1). Therefore, we estimate nutrient concentration from potential temperature *θ* using the linear regression, $C_{{NO_{3}^{ - } }}$ = (− 0.540 ± 0.085) × *θ* + (14.077 ± 1.881) and $C_{{PO_{4}^{3 - } }}$ = (− 0.032 ± 0.006) × *θ* + (0.819 ± 0.138) where error estimates are based on 95% confidence limits using a Student’s *t* test (Fig. S1). Because we are interested in vertical turbulent nutrient flux from the lower layer to the euphotic zone, we used the linear regression coefficients taken in the range where potential temperature is > 15 °C, which roughly corresponds to 400-m or shallower depths. It is worth noting that the regression at L4 is slightly different from those at the other stations. As mentioned in the Results section, turbulence intensity was especially strong at L4, and temperature profiles at L4 were slightly different from the other stations (Fig. 2b). Therefore, the linear regression between nutrients and temperature at L4 was calculated separately: $C_{{NO_{3}^{ - } }}$ = (− 0.939 ± 0.380) × *θ* + (24.666 ± 8.776), $C_{{PO_{4}^{3 - } }}$ = (− 0.065 ± 0.031) × *θ* + (1.720 ± 0.719). Elevated turbulence intensity was also detected at L3, but the high value was found at depths shallower than 100 m, where nutrients were largely depleted.

### Estimation of in situ chlorophyll *a* concentration from fluorescence

A fluorescence meter on the TM profiler enables us to see the vertical distribution of chlorophyll *a* along the section; the fluorescence must be converted to the absolute value of chlorophyll *a* from bottle samples using a shipboard CTD Rosette sampling system. It is not possible to obtain water samples and microstructure measurements simultaneously. In this study, the fluorescence measured by the SBE-9plus on the CTD system was calibrated using chlorophyll *a* concentration from bottle samples, and the fluorescence from the measurement by the TM profiler was calibrated by the calibrated SBE-9plus fluorescence at the same station. Most of the bottle samplings were made during daytime. To minimize effects on the calibration from non-photochemical quenching processes (e.g. Ref.^41^,), we used data collected at ~ 100-m or deeper depths (100, 200, 300, and 400-m). Although there were differences in time (~ 1 h) and measurement position between the CTD and TM casts, the fluorescence obtained with the TM profiler exhibits a good linear correlation with the SBE-9plus fluorescence at stations L2–L4 (Fig. S2a). The linear regression is Flu_SBE_ = (1.42 ± 0.09) × Flu_TM_ + (0.10 ± 0.01) where Flu is fluorescence. At stations L1, L4, and L11, chlorophyll *a* concentration was analyzed with water samples and was utilized to calibrate the SBE-9plus fluorescence (Fig. S2b). Their linear regression is Chl.a = (1.11 ± 0.25) × Flu_SBE_ – (0.08 ± 0.05).

## Supplementary information

Supplementary Figures.

## Data Availability

The data presented in this paper are available at https://ocg.aori.u-tokyo.ac.jp/omix/Tsutsumi_etal_2020SREP.
