# Supplementary material for: Vertical fluxes of nutrients enhanced by strong turbulence and phytoplankton bloom around the ocean ridge in the Luzon Strait
Source: Sci Rep. 2020 Oct 21;10:17879. doi: 10.1038/s41598-020-74938-5 (PMC7577972; doi:10.1038/s41598-020-74938-5)
Supplement: Supplementary file 1 — Supplementary Figures. [file 41598_2020_74938_MOESM1_ESM.pdf]

# Vertical fluxes of nutrients enhanced by strong turbulence and phytoplankton bloom around the ocean ridge in the Luzon Strait

**Eisuke Tsutsumi[1,2,*], Takeshi Matsuno[2,+], Sachihiko Itoh[1,+], Jing Zhang[3,+], Tomoharu Senjyu[2,+], Akie Sakai[4,+], Keunjong Lee[1,+], Daigo Yanagimoto[1,+], Ichiro Yasuda[1,+], Hiroshi Ogawa[1,+], Cesar Villanoy[5,+]**

[1]Atmosphere and Ocean Research Institute, University of Tokyo, Kashiwa, 277-8564, Japan

[2]Research Institute for Applied Mechanics, Kyushu University, Kasuga, 816-8580, Japan

[3]Graduate School of Science and Engineering, University of Toyama, Toyama, 930-8555, Japan

[4]Interdiciplinary Graduate School of Engineering Science, Kyushu University, Kasuga, 816-8580, Japan

[5]Marine Science Institute, University of the Philippines, Metro Manila, 1101, Philippines

[*]tsutsumi@aori.u-toyko.ac.jp

[+]these authors contributed equality to this work

## Supplementary information

This supplementary information includes figures relating temperature to nutrients and chlorophyll *a* data.

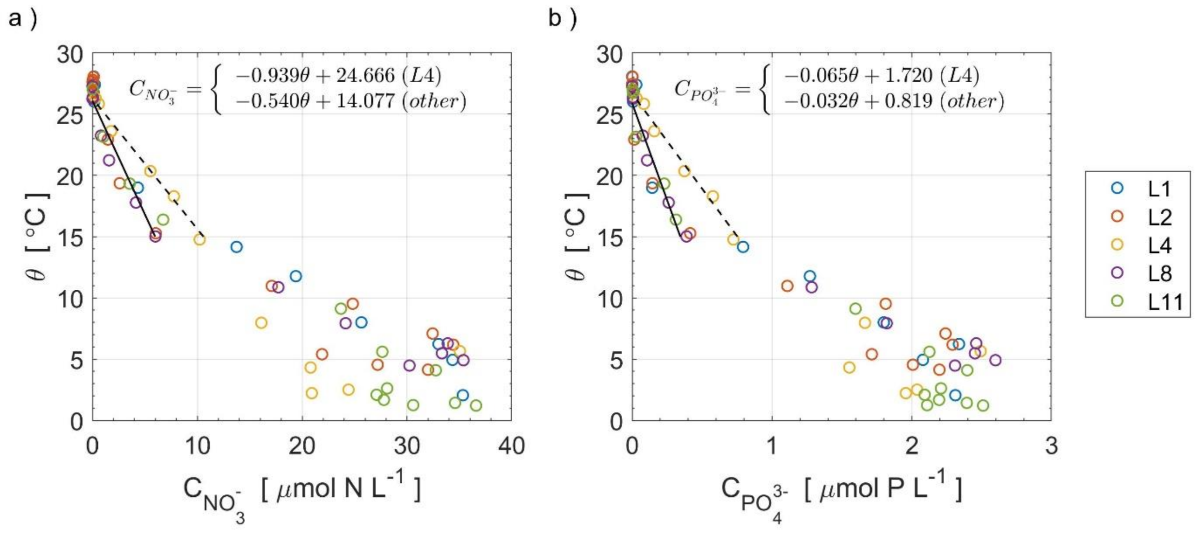


Figure S 1. Relationship between nutrient concentration and potential temperature obtained from CTD casts and bottle water sampling at staions L1, L2, L4, L8, and L11: (a) nitrate and (b) phosphate. Dashed (solid) lines show the linear regression calculated from the data within a range of potential temperatures between 15–27 °C for station L4 and (other stations).

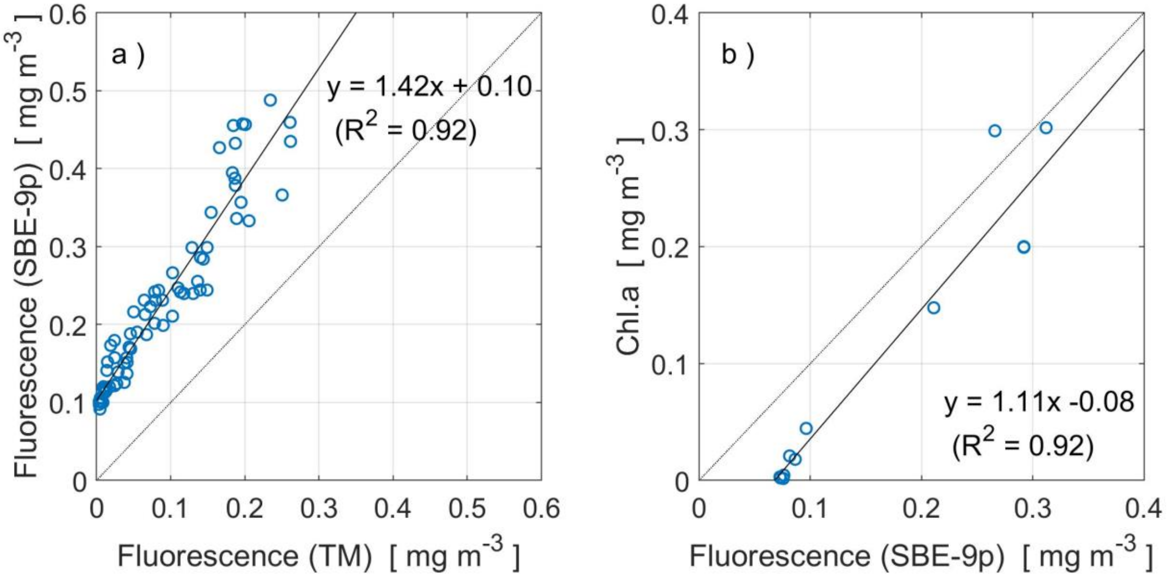


Figure S2. (a) Comparison of fluorescence obtained by TurboMAP (TM) and SBE-9plus. (b) Relationship between fluorescence obtained by SBE-9plus and chlorophyll *a* analyzed with water samples.
